# Paradoxical Leadership and Employee Task Performance: A Sense-Making Perspective

**DOI:** 10.3389/fpsyg.2021.753116

**Published:** 2021-12-16

**Authors:** Wei Zhang, Shudi Liao, Jianqiao Liao, Quanfang Zheng

**Affiliations:** ^1^School of Management, Huazhong University of Science and Technology, Wuhan, China; ^2^Business School, Hubei University, Wuhan, China; ^3^Hubei Center for Studies of Human Capital Development Strategy and Policy, Key Research Base of Humanities and Social Science of Hubei Province, Wuhan, China; ^4^School of Business Administration, Zhongnan University of Economics and Law, Wuhan, China

**Keywords:** paradoxical leadership, sense-making, adaptability, task performance, *Zhong Yong* thinking, organizational identification

## Abstract

Paradoxical leadership has received increasing research attention in recent years. Yet, questions remain as to why and when paradoxical leadership is effective in promoting employee work outcomes. Drawing upon the sense-making perspective, we propose that paradoxical leadership enhances employee task performance by increasing employees’ adaptability, and paradoxical leadership is more effective when employees have higher levels of *Zhong Yong* thinking and organizational identification. To test our hypotheses, we conducted a multi-source and multi-wave survey study among 235 employees and their supervisors in southern China. The results of the regression analyses fully support our hypotheses. In general, our findings shed light on the underlying mechanisms, as well as the boundary conditions, of the effect of paradoxical leadership. The theoretical and practical implications of these findings are discussed.

## Introduction

The concept of paradoxical leadership, defined by Zhang et al. (2015), refers to leaders’ seemingly competing yet interrelated behaviors to meet competing workplace demands simultaneously and over time. This kind of leadership has been one of the most popular research topics in the past few years. Paradoxical leadership attracts such attention because of its advantages in dealing with organizational paradoxes. Evidence has shown that paradoxical leadership is associated with better employee and team performance, such as work role performance, creativity, and innovative behaviors (Zhang et al., 2015, 2021; Shao et al., 2019; Yang et al., 2021).

Employee task performance—reflects to what extent employees achieve the officially required outcomes and behaviors that directly serve the organization’s goals (Motowidlo and Van Scotter, 1994)—is crucial for organizational survival and growth in a complicated and conflicted environment. The competitive pressures which extend from this environment accentuate the importance of achieving superior task performance. Therefore, how to promote individuals’ task performance has become an increasing concern for paradoxical leaders. Many mechanisms have been proposed to explain the positive effects of paradoxical leadership on employees’ task performance, but few studies have been focused on the explanatory mechanisms from the lens of followers’ own interpretations of leader behaviors. The success of a paradoxical leader’s competing value framework is highly dependent on employees’ own interpretations. For example, when tensions from conflicting or competing demands become salient, employees may respond positively or negatively. A negative reaction involves defensive mechanisms such as denial and repression (Vince and Broussine, 1996) that can lead to potentially detrimental outcomes (Lewis, 2000). A positive reaction focuses on opportunity rather than threat and tries to manage the tensions (Miron-Spektor et al., 2018; Sparr, 2018).

In this study, grounded in a sense-making perspective, we develop a theoretical model that describes why and under what conditions paradoxical leadership may affect employees’ task performance. According to sense-making theory, when individuals are faced with a situation, they try to comprehend it by creating their own explanations and meanings (Weick, 1995), which provide goals and motivations for subsequent actions (Drazin et al., 2008; Madjar et al., 2011). The role of paradoxical leadership is to help individuals interpret the competing demands positively (Miron-Spektor et al., 2018) and seek a synthesis method to improve outcomes. Such motivational orientation that may result from the interpretation of a situation is adaptability, which refers to the willingness and ability to change behaviors, feelings, and thoughts in response to environmental demands (McArdle et al., 2007). We predict that paradoxical leadership stimulates employees’ task performance by increasing their adaptability.

Moreover, the explanations and meanings of situations are formed by an interactive combination of cultural values and individual identity (Weick, 1995; Weick et al., 2005; Drazin et al., 2008). *Zhong Yong* thinking represents a cognitive style that explains how the Chinese evaluate and process information, approach tasks, and make decisions (Yang, 2010). It enables individuals to process external information and integrate it with their internal need to take appropriate actions. Employees with *Zhong Yong* thinking are more likely to align their cognitions and behaviors with those of paradoxical leaders. Organizational identification is a form of collective work identity, referring to incorporating a group’s beliefs and values into one’s own identity and self-image (Pratt, 1998), which may affect individuals’ information interpretation and motivation of actions. Accordingly, we examine *Zhong Yong* thinking and organizational identification as moderators that influence the relationship between paradoxical leadership and employee task performance.

This study makes several contributions to the literature. First, beyond commonly used theories for paradoxical leadership, such as paradox theory (e.g., Shao et al., 2019), yin-yang philosophy (e.g., Zhang et al., 2015), social learning theory (e.g., Ishaq et al., 2021), and self-determination theory (Yang et al., 2021), we introduce sense-making theory to broaden our understanding of the association between paradoxical leadership and employees’ outcomes. According to sense-making theory, we argue that when leaders behave paradoxically in order effectively to manage demands that are not isolated but are inherently interrelated and in conflict with one another for the survival of the organization (Smith et al., 2012), they attempt to trigger organizational members’ sense-making about what their jobs entail and how to do them (Lüscher and Lewis, 2008).

Second, we address the question of why paradoxical leadership is associated with task performance. When leaders engage in sense-giving, followers are not simply passive recipients of meaning but instead engage in their sense-making and adapt, alter, resist or reject the sense they have been given (Pratt, 2000; Sonenshein, 2010). However, the interpretations and efforts of employees have been overlooked, which highlights the necessity of examining adaptability as a mediating mechanism in our study. In addition, adopting a perspective of sense-making in our study into the role of employee adaptability not only explains why paradoxical leadership enhances task performance but also translates the impact of paradoxical leadership to real outcomes.

Third, we propose and test the interactive effects of paradoxical leadership, *Zhong Yong* thinking, and organizational identification on adaptability and subsequent employee outcomes in the workplace. Our study contributes to the literature by identifying boundary conditions of the effect of paradoxical leadership. In doing so, we suggest that individual cultural values, such as *Zhong Yong* thinking, and work identity, such as organizational identification, may help them respond positively to the complex behavior of leaders and display high levels of task performance.

## Theoretical Background and Hypothesis Development

### Paradoxical Leadership

Paradoxical leadership is characterized by leader behaviors that are seemingly competing yet interrelated to meet competing workplace demands simultaneously and over time (Zhang et al., 2015). It requires leaders to reframe their thinking about contradictions from ‘either/or’ to ‘both/and’ (Smith and Berg, 1987). Paradoxical leadership is manifested in five pairs of contradictory behaviors (Zhang et al., 2015): (1) leader displays a combination of self-centerdness and other-centerdness when he or she maintains their central influence while simultaneously sharing recognition and leadership with followers. (2) Maintaining both distance and closeness is shown when a leader maintains hierarchical distinctions when dealing with work-related issues while simultaneously forming close relationships privately with subordinates. (3) Treating subordinates uniformly while allowing individualization is shown when a leader balances uniformity and individualization, such as assigning the same workloads, while simultaneously allocating different parts of the work based on individuals’ skills or interests. (4) Enforcing work requirements while allowing flexibility is shown when a leader controls subordinates’ behaviors and output while giving them the freedom to deal with problems flexibly and autonomously. (5) Finally, maintaining control over decisions while allowing autonomy, similar to enforcing work requirements while allowing flexibility, emphasizes the balance between control and empowerment.

Most research on paradoxical leadership has focused on its beneficial influence in organizations, as exemplified by improvements in performance (Smith and Lewis, 2011; Amason, 2017), creativity (Knight and Harvey, 2015), commitment (Smith, 2015), competitiveness (Fredberg, 2014; Derksen et al., 2017), the workplace environment (Lewis and Smith, 2014; Gnyawali et al., 2016; Knight and Paroutis, 2017) and career success (Derksen et al., 2017). In addition to its beneficial effects on organizations, research has shown that paradoxical leadership has positive influences on employees in terms of their work attitudes (Kan and Parry, 2004; Garg, 2016), work engagement (Alfes and Langner, 2017; Fürstenberg et al., 2021), work role performance (Zhang et al., 2015), creativity (Shao et al., 2019; Yang et al., 2021), and innovative behaviors (Milosevic et al., 2015; Ingram et al., 2016; Zhang et al., 2021). In summary, paradoxical leadership is widely considered to be an effective leadership style for managing the complex environments faced by modern organizations (Smith and Lewis, 2011; Zhang et al., 2015; Miron-Spektor et al., 2018).

### Paradoxical Leadership and Employee Task Performance

Task performance refers to the role-prescribed activities of an employee (Borman and Motowidlo, 1993). It pertains to individuals’ involvement in accomplishing assigned tasks by an enterprise (Motowidlo, 2000). Task performance is the foundation for organizations to survive. Leadership is a critical situational factor that affects employees’ task performance (Hocine and Zhang, 2014). According to the sense-making theory, sense-making within an organization is related to understanding and is cognitive in nature (Gioia and Chittipeddi, 1991). Previous research on leadership has highlighted the importance of situation-specific cognition, which supports sense-making and leadership as well as its influence on subordinates’ outcomes (Lord and Hall, 2005; Mumford et al., 2007). In a complex and dynamic context, sense-making is seen as a key leadership capability (Ancona, 2011). The role of leaders is to serve as sense-givers. ‘Sense-giving-for-others’ is the process of making sense of this complex situation by themselves, then disseminating new understanding to subordinates to influence their ‘sense-making-for-self’ and subsequent devising a resolution to any situation (Foldy et al., 2008). Drawing on this argument, we suggest that paradoxical leaders may enhance employees’ task performance by creating an environment that accepts contradiction as natural and persistent. Employees who make sense of paradoxical leader behavior may see conflicting and competing demands as intrinsic phenomena in organizations (Smith and Lewis, 2011; Miron-Spektor et al., 2018) and may bring different demands together such that the contradiction between them may transform into productive rather than intractable (Smith et al., 2012; Ingram et al., 2016). By serving as role models, paradoxical leaders allow their followers to observe how to deal constructively with paradoxical situations. Employees may work more confidently and purposefully by observing and modeling the leader’s behaviors. Simultaneously, a paradoxical leader also provides support to reduce employees’ paradox-related uncertainty (Maitlis and Sonenshein, 2010). For example, a leader may encourage employees to use new methods to increase output while accepting the possibility of failure. Taking these observations together, we propose the following hypotheses:

**Hypothesis 1:** Paradoxical leadership is positively related to employees’ task performance.

### The Mediating Role of Employees’ Adaptability

The sense-making theory proposes that sense-making is triggered by failure to confirm one’s self (Weick, 1995). Individuals construct their roles through ways that meet their needs for self-enhancement, self-efficacy and, self-consistency (Erez and Earley, 1993). When individuals feel their role in the organization threatened, they are triggered to engage in sense-making around the source of threat and take actions to recover their role (Maitlis and Christianson, 2014). An important characteristic of sense-making is that it is *based on extracted cues* (Weick, 1995).

When facing a paradox, one fundamental role of a leader is to foster employee intrinsic motivation to build commitment and excitement for work (Andriopoulos and Lewis, 2009). We suggest that one way that paradoxical leaders influence employee task performance is through employee adaptability. Individual adaptability is one’s ability, skill, disposition, willingness, or motivation to change or fit different the task, social, and environment features (Ployhart and Bliese, 2006). Adaptability is a positive motivational orientation toward changing oneself (Wang et al., 2017). Therefore, one of the biggest challenges facing leaders is to enable employees to adapt in the face of an increasingly dynamic and demanding environment (Uhl-Bien and Arena, 2018). From this perspective, a paradoxical leader’s role is to sense and shape opportunities and threats, and expect employees to adapt in accordance with their environment. Moreover, paradoxical leaders create supportive contexts in which individuals choose how and where to focus their energies (Uhl-Bien and Arena, 2018). For example, a paradoxical leader can communicate with subordinates to reduce their feelings of anxiety, uncertainty, and threat (Vince and Broussine, 1996; Lewis, 2000; Schad et al., 2016). A paradoxical leader also has a sharing attitude, which can influence subordinates’ motivation willingness to adapt.

Adaptable employees have been found to exhibit less anxiety (Lewis and Smith, 2014) and to deal creatively with change (Miron-Spektor et al., 2011). Adaptable employees are also more likely to maintain positive affect and constructive behavior, even in uncertain situations (Sparr, 2018). Researchers have found that adaptable teams are more likely to generate new and innovative ideas (Axtell et al., 2000) and engage in job-crafting behaviors (Wang et al., 2017). In terms of task performance, we argue that adaptable employees may be motivated to act spontaneously to cope with paradoxes. Miron-Spektor et al. (2018) demonstrated that employees with a paradox mindset could help them to improve in-role job performance and innovation.

Taken together, we propose the following hypotheses:

**Hypothesis 2:** Employees’ adaptability mediates the relationships between paradoxical leadership and task performance.

### The Moderating Role of *Zhong Yong* Thinking

Due to different historical traditions and regional cultures, Chinese people have great differences in thinking mode from Westerners. Chinese people usually look at problems from a holistic and dialectical point of view, while Westerners deal with problems analytically and pay attention to the characteristics of things themselves. Originating from Confucian philosophy, *Zhong Yong* thinking, also known as the doctrine of the mean, is defined as a cognitive style that requires individuals to consider things from different perspectives, recognize broader situations, avoids going to extremes, and maintain harmony (Ji et al., 2010). According to Wu and Lin (2005), *Zhong Yong* thinking consists of three features: holistic thinking, perspective integration and harmony maintenance. Holistic thinking refers to how individuals recognize things from different aspects; thus, holistic thinking can promote individuals to consider situations from a wide range of views so they can adjust to contradictory situations (Pan and Sun, 2018). Perspective integration refers to integrating of one’s own opinion with those of others and seeking solutions that are acceptable to all by adopting compromising approaches to discussing problems (Ji et al., 2010). Harmony maintenance refers to developing harmonious relationships with others. A harmonious relationship demands that individuals understand the other’s behavior and subsequently adjust one’s own behavior (Pan and Sun, 2018). Therefore, *Zhong Yong* thinking offers an alternative cognitive style. By adopting a *Zhong Yong* thinking, individuals not only perceive and adjust their inner selves, but also change their behavior according to the different external environment (Wu and Lin, 2005). For the external environment, *Zhong Yong* thinkers tend to consciously process the information they hear and integrate it with their internal needs, then choose the most appropriate behavior. This coincides with one of the characteristics of sense-making; that is, sense-making concerns the action that individuals take to make sense of a situation (Maitlis and Christianson, 2014). Individuals with *Zhong Yong* thinking make a continuous effort to understand connections among peoples, places, and events.

Employees receive contradictory and interdependent demands from their paradoxical leaders. Product developers, for example, are asked to consider cost issues and strictly follow specifications when developing new products. If employees have *Zhong Yong* thinking, they may have different interpretations, sensitivity and flexibility regarding the demands conveyed by a paradoxical leader. They may also think about how to integrate the leader’s demand with their abilities and take appropriate action. As individuals with high *Zhong Yong* thinking seek a compromise between extremes (Yao et al., 2010), they will try to select cooperative strategies and compromises to stay consistent with their leaders’ cognitions and behaviors. Subordinates of paradoxical leaders learn to increase their capacity to respond to a changing environment (Detert and Burris, 2007). The greater the *Zhong Yong* thinking of employees, the more a paradoxical leader’s values, goals and norms will be internalized into their role cognitions and behaviors. In addition, due to the principle of holism, individuals with high *Zhong Yong* thinking will seek to deal creatively with change while simultaneously maintaining their efficiency (Miron-Spektor et al., 2011).

Therefore, we propose that *Zhong Yong* thinking enables employees to be more flexible and open to be consistent with paradoxical leadership, thereby becoming more actively accepting paradoxical leaders’ efforts to achieve the best possible task performance.

**Hypothesis 3 (H3):** *Zhong Yong* thinking moderates the relationship between paradoxical leadership and employees’ adaptability, such that the positive relationship is stronger for employees with high *Zhong Yong* thinking than for those with low *Zhong Yong* thinking.

Taking these hypotheses together, a moderated mediation model is formed in which the relationship between paradoxical leadership and subordinates’ adaptability and the mediated relationship between paradoxical leadership and subordinates’ task performance depending on the level of subordinates’ *Zhong Yong* thinking. In other words, we propose that paradoxical leaders improve task performance by increasing adaptability, which is more likely to occur when individuals have a high level of *Zhong Yong* thinking. This leads to the following hypotheses:

**Hypothesis 4:** Employees’ *Zhong Yong* thinking moderates the mediating effect of adaptability on the relationship between paradoxical leadership and task performance, such that the indirect effect of paradoxical leadership on task performance via adaptability is stronger for high *Zhong Yong* thinking than for low *Zhong Yong* thinking.

### The Moderating Role of Organizational Identification

According to sense-making theory, a main characteristic of sense-making is that it is social, grounded in identity construction (Weick, 1995). sense-making can be difficult and lead to confusion without social roles and relationships within an organization (Weick, 1993). organizational identification represents common attributes that bind the individuals to their organizations (Dutton et al., 1994), which may influence individuals’ issue-interpretation, meaning-creation, and engagement in creative actions toward their organizations (Madjar et al., 2011). And another important feature of sense-making is that it is based on extracted cues (Weick, 1995), which indicates that individuals focus only on one part of their environment. Ford (1996) suggests that individuals tend to favor cues that are consistent with their personality. A paradoxical leader is part of the environment, which will influence employees’ selection of environmental cues for adapting in the face of complex challenges. Therefore, we examine an interactive effect of paradoxical leadership (i.e., cue extraction) and organizational identification (i.e., identity construction) on employee adaptability.

Organizational identification is defined as the individual’s sense of group or belonging to an organization in which an individual defines one’s own identity as a member (Mael and Ashforth, 1992). The more an individual identifies with an organization, the more the organization’s identity is incorporated into the individual’s self-concept (Dutton et al., 1994). Employees with high organizational identification have a higher desire to enhance their self-concept, self-esteem, and self-worth to cope with a changing environment. Moreover, previous research on leadership has found that an employee’s self-concept is a moderating factor in leadership processes (van Knippenberg et al., 2004; Wang et al., 2017). Consisting with this notion, we suggest that employees with high organizational identification can adjust to paradoxical leadership in a more positive way. Therefore, we propose:

**Hypothesis 5 (H5):** Employees’ organizational identification moderates the relationship between paradoxical leadership and employees’ adaptability, such that the positive relationship is stronger for employees with high organizational identification than for those with low organizational identification.

We further suggest that employees’ organizational identification moderates the indirect relationship between paradoxical leadership and task performance. Drawing on our discussion for Hypotheses 1, 2, and 5, we predict that for employees with a high level of organizational identification, paradoxical leadership will enhance adaptability, which in turn will promote performance outcomes. In contrast, for those with a low level of organizational identification, paradoxical leadership will hinder task performance by reducing adaptability.

**Hypothesis 6:** Employees’ organizational identification moderates the mediating effect of adaptability on the relationship between paradoxical leadership and task performance, such that the indirect effect of paradoxical leadership on task performance via adaptability is stronger for high organizational identification than for low organizational identification.

**FIGURE 1:**
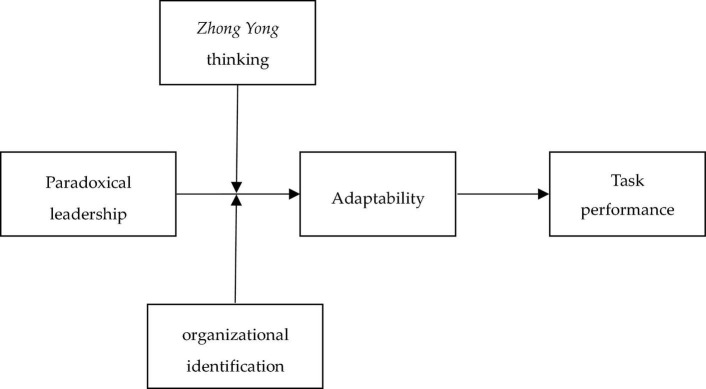
Conceptual model.

The model we propose is illustrated in Figure 1.

## Materials and Methods

### Sample and Procedure

To test our hypotheses, we used a multi-respondent cross-sectional survey design to collect data. Data were collected from full-time employees, including employees and their immediate supervisors in 10 companies in southern China. These companies were all from high-tech industries. The research assistants handed out dyadic questionnaires that were filled out separately by supervisors and their subordinates, with the help of the human resource departments of each company. The covering letter of the questionnaires indicated that their data would be kept completely confidential and used only for scientific research.

Two separate surveys were administered for 1 month in order to reduce the influence of homologous error. At Time 1, the employees rated paradoxical leadership, adaptability, *Zhong Yong* thinking, and organizational identification. One month later, at Time 2, the supervisors rated their subordinates’ task performance. At Time 1, a total of 315 employees filled in the questionnaire. At Time 2, a total of 268 supervisors filled in the questionnaire. After removing invalid responses, we had 235 unique supervisor-subordinate dyads who had completed questionnaires; the response rate was 74.6%.

Among the participates, 159 (67.7%) supervisors and 127 (54.0%) subordinates were male. The mean age of supervisors was 36.09 years (*SD* = 5.73) and of subordinates 30.34 years (*SD* = 5.47). About 95.3% of supervisors and 82.5% of subordinates had college or higher degrees. On average, supervisors had 5.29 years (*SD* = 2.86) of work experience in their current company, and subordinates on average had worked for 3.89 years (*SD* = 4.45) in their current company.

### Ethical Statement

Before starting data collection, ethical approval was applied and approved by the Academic Committee of the University. All procedures performed in studies involving human participants did not violate any legal regulations or common ethical guidelines. In order to ensure that ethical principles are followed in this study, the purpose of this research was introduced and informed consent was obtained from all individual participants included in the study. Moreover, all participants were assured that they could reject any questions or withdraw from the survey at any time. Lastly, individual participants’ anonymity and confidentiality were assured.

### Measures

#### Paradoxical Leadership

We used the twenty-two items developed by Zhang et al. (2015) to measure paradoxical leadership behavior. The scale contains five dimensions: combining self-centerdness with other-centeredness; maintaining both distance and closeness; treating subordinates uniformly while allowing individualization; enforcing work requirements while allowing flexibility; and maintaining decision control while allowing autonomy. Employees were asked to rate their leader’s paradoxical leadership behavior. A sample item was ‘Uses a fair approach to treat all subordinates uniformly, but also treats them as individuals.’ All items were rated on a 5-point Likert scale ranging from ‘strongly disagree’ (1) to ‘strongly agree’ (5). Cronbach’s α of five dimensions were UI = 0.86, SO = 0.83, CA = 0.89, RF = 0.85, DC = 0.88.

#### Adaptability

We used the nine items developed by Van der Heijde and Van der Heijden (2006) and shortened by Van der Heijden et al. (2018) to measure subordinates’ adaptability. According to Van der Heijde and Van der Heijden (2006), subordinates’ adaptability contains two dimensions: anticipation and optimization, and personal flexibility. Anticipation and optimization were measured with four items. A sample item was ‘I consciously devote attention to applying my newly acquired knowledge and skills.’ Personal flexibility was measured with five items. A sample item was ‘I adapt to developments within my organization.’ All items were rated on a 5-point Likert scale ranging from ‘strongly disagree’ (1) to ‘strongly agree’ (5). Cronbach’s α of two dimensions were AO = 0.85, PF = 0.83.

#### Task Performance

We used the three items developed by Farh et al. (1991) to measure task performance. Supervisors were asked to rate their direct subordinates’ task performance from three aspects: work quality, work efficiency and the completion of the work target. A sample item was ‘Are your subordinate’s work outcomes perfect, free of error and of high accuracy?’ All items were rated on a 5-point Likert scale ranging from ‘strongly disagree’ (1) to ‘strongly agree’ (5). Cronbach’s α was 0.79.

#### *Zhong Yong* Thinking

We used the thirteen items developed by Wu and Lin (2005) to measure *Zhong Yong* thinking. The scale contains three dimensions: holistic thinking, perspective integration and harmony. The participants were asked to report their own *Zhong Yong* thinking. A sample item was ‘I try to find a balance between my own opinions and those of others.’ All items were rated on a 5-point Likert scale ranging from ‘strongly disagree’ (1) to ‘strongly agree’ (5). Cronbach’s α of three dimensions were HT = 0.89, PI = 0.86, HM = 0.87.

#### Organizational Identification

We used the five items developed by Smidts et al. (2001) to measure employees’ organizational identification. Employees were asked to report their own organizational identification. A sample item was ‘I experience a strong sense of belonging to my company.’ All items were rated on a 5-point Likert scale ranging from ‘strongly disagree’ (1) to ‘strongly agree’ (5). Cronbach’s α was 0.94.

#### Control Variables

For assessing predictive validity, we considered several relevant control variables, such as gender (0 = female, 1 = male), and educational level (1 = high school degree or less, 2 = practical degree, 3 = bachelor’s, 4 = master’s or higher). We also controlled for the organizational tenure of the supervisors and employees, both of which might have an influence on the interaction between them.

## Results

### Confirmatory Factor Analysis

We used Amos 21.0 to conduct confirmatory factor analysis (CFA) to test the factorial validity of our measures through maximum likelihood estimation. The measurement model consisted of all measured variables, including paradoxical leadership (UI, treating subordinates uniformly while allowing individualization; SO, combining self-centeredness with other-centeredness; CA, maintaining decision control while allowing autonomy; RF, enforcing work requirements while allowing flexibility; DC, maintaining both distance and closeness), adaptability (AO, anticipation and optimization; PF, personal flexibility), *Zhong Yong* thinking (HT, holistic thinking; PI, perspective integration; HM, harmony), organizational identification, task performance and creativity. A total of variables/dimensions of were taken as latent variables, and the items of each variable/dimension were taken as explicit variables. The reliability and validity of our study questionnaire were evaluated by calculating composition reliability (CR) and average variance extracted (AVE) through the factor loading of CFA.

For this study, the standardized factor loading of the variables/dimensions was between 0.529 and 0.929. Cronbach’s α ranged from 0.789 to 0.944. The CR ranged from 0.791 to 0.945 and the AVE was between 0.511 and 0.775 (see Table 1). They were all in line with the recommended values given by Fornell and Larcker (1981): the standardized factor loading was greater than 0.5, the AVE should be greater than 0.5, and the CR was greater than 0.6. The test results of discriminate validity showed that each square root of AVE ranged from 0.715 to 0.880 (see Table 1), which is greater than the correlation coefficient between the constructs and meets the criteria suggested by Fornell et al. (1982). This finding indicates that the questionnaire developed in this study had high internal consistency reliability, composition reliability, convergence validity and discriminant validity.

**TABLE 1:** Internal consistency reliability, composition reliability, convergent validity, and discriminant validity.

Variables	Factor	Item	Factor loading	Reliability		Convergent validity	Discriminant validity	
				α	CR	AVE	r	Square root of AVE
Paradoxical leadership	UI	PL1∼PL5	0.559∼0.858	0.856	0.864	0.564	0.080∼0.762	0.751
	SO	PL6∼PL10	0.559∼0.811	0.832	0.839	0.515	0.094∼0.762	0.718
	CA	PL11∼PL14	0.737∼0.869	0.892	0.893	0.677	0.137∼0.614	0.823
	RF	PL15∼PL18	0.735∼0.803	0.852	0.854	0.594	0.103∼0.604	0.771
	DC	PL19∼PL22	0.765∼0.835	0.875	0.877	0.642	0.098∼0.604	0.801
Zhong Yong thinking	HT	ZY1∼ZY4	0.772∼0.899	0.887	0.888	0.666	0.077∼0.760	0.816
	PI	ZY5∼ZY9	0.564∼0.826	0.856	0.861	0.558	0.094∼0.831	0.747
	HM	ZY10∼ZY13	0.687∼0.819	0.871	0.876	0.640	0.080∼0.831	0.800
Organizational identity	OI	OI1∼OI5	0.799∼0.929	0.944	0.945	0.775	0.204∼0.914	0.880
Adaptability	AO	AD1∼AD4	0.618∼0.860	0.853	0.857	0.603	0.195∼0.914	0.777
	PF	AD5∼AD9	0.529∼0.836	0.833	0.836	0.511	0.110∼0.360	0.715
Task performance	TP	TP1∼TP3	0.727∼0.761	0.789	0.791	0.557	0.077∼0.396	0.746

In order to control the common-method variance, we collected data at two time points, since the independent variable ‘paradoxical leadership’ and the mediator ‘adaptability’ were answered by employees at the same time, the problem of the homologous variance may exist. So we adopted method such as concealment of survey content and information of respondents, setting reverse questions, and different scales (Likert-5, Likert-7) to reduce response tendency in the survey. The fit indices of the CFA (χ^2^ = 1736.608, df = 1208, CFI = 0.931, TLI = 0.924, RMSEA = 0.043, SRMR = 0.049) are all acceptable (lower than 0.95), indicating that the confirmatory factor model we constructed is scientific and reasonable (see Table 2).

**TABLE 2:** Indices of model fit.

Index of model fit	χ^2^	df	χ^2^/df	CFI	TLI	RMSEA	SRMR
	–	–	<3	>0.9	>0.9	<0.08	<0.08
Results	1736.608	1208	1.438	0.931	0.924	0.043	0.049

The means, standard deviations and correlations are shown in Table 3. The results showed that paradoxical leadership was positively related to adaptability (*r* = 0.33, *p* < 0.01) and task performance (*r* = 0.28, *p* < 0.01). Thus, our hypotheses received preliminary support.

**TABLE 3:** Descriptive statistics and correlation coefficient matrix.

Variables	*M*	*SD*	Correlations						
			1	2	3	4	5	6	7
(1) Gender	0.68	0.47	1						
(2) Organizational Tenure (year)	5.29	2.86	0.00	1					
(3) Education	3.26	0.55	–0.10	0.05	1				
(4) PL	3.87	0.48	–0.01	0.08	0.08	1			
(5) ZYT	2.13	0.63	–0.03	0.04	–0.04	0.17**	1		
(6) Organization Identification	3.91	0.82	–0.03	–0.01	–0.06	0.32**	0.35**	1	
(7) Adaptability	3.87	0.61	–0.03	–0.03	0.07	0.33**	0.26**	0.33**	1
(8) Task performance	3.99	0.72	–0.03	–0.06	–0.05	0.28**	0.09	0.09	0.32**

*n = 235. 0 = female; 1 = male; PL, paradoxical leadership; ZYT, Zhong Yong thinking.*

*p < 0.05, **p < 0.01.

### Hypothesis Testing

We adopted a stepwise regression method to test our hypotheses. The results are shown in Table 4. First, the results of Model 9 indicated that paradoxical leadership had a positive relationship with task performance (β = 0.29, *p* < 0.001); the total effect of paradoxical leadership on task performance was significant. H1 was supported; Second, Model 2 indicated that paradoxical leadership had a positive relationship with adaptability (β = 0.33, *p* < 0.001); Third, Model 10 indicated that paradoxical leadership and adaptability had a positive relationship with task performance (β = 0.21, *p* < 0.01; β = 0.26, *p* < 0.001). The results indicated that adaptability plays a partial mediating role in the relationship between paradoxical leadership and task performance. To further test the mediating effect of adaptability, we adopted the test method proposed by Preacher et al. (2007), using bootstrapping with the SPSS Process. The results of bootstrapping showed that the indirect effect was significant (a × b = 0.130), 95% confidence interval [LLCI = 0.060, ULCI = 0.220]. Therefore, H2 was supported.

**TABLE 4:** Analysis of regression.

	Adaptability							Task performance		
	M1	M2	M3	M4	M5	M6	M7	M8	M9	M10
Gender	–0.02	–0.02	–0.01	0.00	–0.01	0.01	0.02	–0.03	–0.03	–0.03
Tenure(year)	–0.03	–0.06	–0.06	–0.05	–0.05	–0.07	–0.01	–0.06	–0.08	–0.07
Education	0.06	0.04	0.05	0.06	0.06	0.05	0.07	–0.05	–0.07	–0.08
PL		0.33***	0.29***	0.28***	0.25***	0.28***	0.20***		0.29***	0.21**
ZYT			0.22**	0.20**			0.11**			
OI					0.25***	0.27***	0.04			
Adaptability										0.26***
PLxZYT				0.24***			0.16***			
PLxOI						0.32***	0.13***			
ZYTxOI							−0.18***			
PLxZYTxOI							–0.02			
*R* ^2^	0.01	0.11	0.16	0.21	0.17	0.27	0.35	0.01	0.09	0.15
Δ*R*^2^		0.11***	0.05**	0.06***	0.06***	0.10***	0.35***		0.08***	0.06***
*F*	0.44	7.40***	8.66***	10.33***	9.29***	14.16***	17.32***	0.52	5.68***	7.99***

*n = 235.*

*PL, paradoxical leadership; ZYT, Zhong Yong thinking; OI, organizational identification.*

*p < 0.05, **p < 0.01, ***p < 0.001.

**FIGURE 2:**
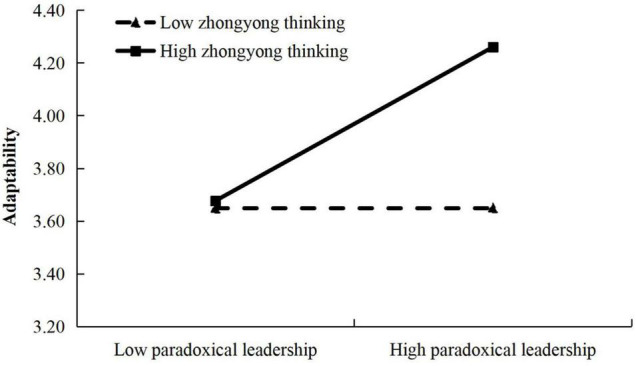
Interaction effect of paradoxical leadership and *Zhong Yong* thinking.

To examine the moderating effect of *Zhong Yong* thinking and organizational identification, we followed the suggestion of Cohen et al. (2003). We first centralized the independent variable ‘paradoxical leadership,’ the moderator variables ‘*Zhong Yong* thinking’ and ‘organizational identification,’ and then respectively constructed the interaction items ‘paradoxical leadership’ and ‘*Zhong Yong* thinking,’ and ‘paradoxical leadership’ and ‘organizational identification.’ As shown in Table 4, Model 2 indicated that paradoxical leadership had a positive relationship with adaptability (β = 0.33, *p* < 0.001). In Model 4, we put ‘paradoxical leadership’ and ‘*Zhong Yong* thinking’ into the regression equation model at the same time, and results showed that paradoxical leadership still had a positive relationship with adaptability (β = 0.28, *p* < 0.001), and the interaction between ‘paradoxical leadership’ and ‘*Zhong Yong* thinking’ had a positive relationship with adaptability (β = 0.24, *p* < 0.001). To better understand the moderating effect, according to the suggestion of Cohen et al. (2003), we used Process program by Hayes and Usami (2020)^1^ to conduct a simple slope test. Figure 2 shows that paradoxical leadership was more positively related to adaptability when *Zhong Yong* thinking was high than when it was low. Therefore, H3 was supported.

**FIGURE 3:**
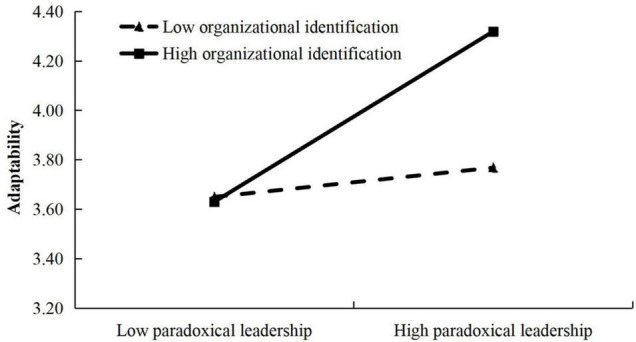
Interaction effect of paradoxical leadership and organizational identification.

We further tested the moderating effect of organizational identification. On the basis of Model 2, we put ‘paradoxical leadership’ and ‘organizational identification’ into the regression equation model at the same time (Model 6), and results showed that paradoxical leadership still had a positive relationship with adaptability (β = 0.28, *p* < 0.001), and the interaction between paradoxical leadership and organizational identification had a positive relationship with adaptability (β = 0.32, *p* < 0.001). Figure 3 shows that paradoxical leadership was more positively related to adaptability when organizational identification was high than when it was low. Therefore, H5 was supported.

In order to test the robustness of the results, we controlled for the three-way interaction (and the interactive term of *Zhong Yong* thinking and organizational identification) in the regression analysis (Model 7), results showed that paradoxical leadership still had a positive relationship with adaptability (β = 0.20, *p* < 0.001), and the interaction between paradoxical leadership and *Zhong Yong* thinking, paradoxical leadership and organizational identification still had a positive relationship with adaptability (β = 0.16, *p* < 0.001; β = 0.13, *p* < 0.001).

As shown in Table 5, when *Zhong Yong* thinking was at a low level, the indirect effect of paradoxical leadership on task performance via adaptability was not significant (*effect* = 0.03, 95% CI [–0.04, 0.10]). When *Zhong Yong* thinking was at a high level, the results were significant (*effect* = 0.18, 95% CI [0.09, 0.31]). By comparing the difference mediating effect of each level, we found that confidence interval of mediating effect difference between high and low level did not contain zero (*effect* = 0.16, 95%CI [0.06, 0.30]), indicating the mediating effect of each level was significantly different. Moreover, the moderated mediation effect was 0.12, and the 95% CI was [0.05, 0.24]; neither contained zero. These results indicated that the indirect effect of paradoxical leadership on task performance via adaptability was moderated by *Zhong Yong* thinking. Taken together, H4 was supported.

**TABLE 5:** Moderated mediation results of *Zhong Yong* thinking.

	*Zhong Yong* thinking	Effect	BootSE	95% CI	
				LLCI	ULCI
Conditional indirect effects	Low(–1 SD)	0.03	0.03	–0.04	0.10
	High(+1 SD)	0.18	0.05	0.09	0.31
Difference (High–Low)		0.16	0.06	0.06	0.30
Index of moderated mediation		0.12	0.05	0.05	0.24

Similarly, as shown in Table 6, when organizational identification was at a low level, the indirect effects of paradoxical leadership on task performance via adaptability was not significant (*effect* = 0.0004, 95% CI [–0.05, 0.06]). When organizational identification was at a high level, the results were significant (*effect* = 0.22, 95% CI [0.11, 0.35]). By comparing the difference mediating effect of each level, we found that the confidence interval of mediating effect difference between high and low level did not contain zero (*effect* = 0.22, 95%CI [0.10, 0.34]), indicating the mediating effect of each level was significantly different. Moreover, the moderated mediation effect was 0.13, and the 95% CI was [0.06, 0.21], neither contained zero. These results indicated that the indirect effect of paradoxical leadership on task performance and creativity via adaptability was moderated by organizational identification. Taken together, H6 was supported.

**TABLE 6:** Moderated mediation results of organizational identification.

	Organizational identification	Effect	BootSE	95% CI	
				LLCI	ULCI
Conditional indirect effects	Low(–1 SD)	0.0004	0.03	–0.05	0.06
	High(+1 SD)	0.22	0.06	0.11	0.35
Difference(High–Low)		0.22	0.06	0.10	0.34
Index of moderated mediation		0.13	0.04	0.06	0.21

## Discussion

Based on sense-making theory, we developed and tested a model to examine how paradoxical leadership affects employee performance outcomes via enhancing employee adaptability, and how employee *Zhong Yong* thinking and organizational identification influence the effectiveness of paradoxical leadership. The results of a questionnaire survey of 235 sets of paired data from supervisors and their direct subordinates found that paradoxical leadership promoted employees’ adaptability, thereby improving followers’ performance outcomes. Moreover, the results supported the conditional indirect effects of paradoxical leadership on task performance.

### Theoretical Implications

Despite the fact that paradoxical challenges are pervasive in organizations at both the organization level (Smith and Lewis, 2011; Schad et al., 2016) and the employee level (Zhang et al., 2015; Shao et al., 2019; Fürstenberg et al., 2021), the effectiveness of leaders is becoming a central issue for organizations to embrace inconsistencies and seek sustainable development. Our study makes several contributions to the literature. First, our study extends and adds value to the sense-making theory by linking it with paradoxical leadership. Although a variety of theoretical perspectives—such as paradox theory, yin-yang philosophy, social learning theory and self-determination theory—have been used to explain the impacts of paradoxical leadership on employees’ motivations and behaviors at work, this line of study has mostly focused on what specific leader behaviors can promote adaptability and when. In particular, it reveals that leaders can promote the adaptability of their followers by adopting a “both/and” approach to trigger followers to engage in sense-making around the sources of opportunities and threats and act so as to restore their identity in the face of dynamic and complex environment (Maitlis and Christianson, 2014). Such a leadership approach improves employee adaptability and subsequent task performance depending on individual cultural factors and organizational context factors. Taking together, our findings extend sense-making theory by demonstrating how paradoxical leaders trigger their members’ sense-making about the organizational environment and how to do (Lüscher and Lewis, 2008).

Second, we take a step toward resolving a critical argument about how paradoxical leadership help individuals address task performance outcomes in complicated and conflicted environment. Several studies have recognized that sense-making is initiated when leaders arrive with a vision for the organization in response to environment changes (Ravasi and Schultz, 2006; Nag et al., 2007). Thus, sense-making by individuals occur in response to leader’s sensegiving which shape members’ understanding of a positive way forward. This positive way is the individuals’ willingness to adapt to a leader’s behavior, and to be more flexible and open to be consistent with paradoxical leadership, to overcome challenges and changes. Therefore, we highlight the necessity of examining adaptability as a mediating mechanism in our study.

Third, following the sense-making theory, we contribute to the literature on leadership by extending the boundary conditions under which paradoxical leadership are more effective. Specifically, the empirical results show that two important boundary conditions (*Zhong Yong* thinking and organizational identification) are the two factors that affect the effectiveness of paradoxical leadership. In other words, paradoxical leadership tends to elicit a high level of individual adaptability in dynamic cognitions rather than in stable cognitions in that *Zhong Yong* thinkers tend to consciously process the external information with their internal needs. This is consistent with the suggestion by Miron-Spektor et al. (2018) that the importance of employees’ paradox mindset in responding to organizational paradox is triggered by resource scarcity. Moreover, our treatment of organizational identification as a moderator also makes a contribution to identity literature. We find that the effect of organizational identification is sufficient to make paradoxical leadership conducive to employee adaptability and subsequent performance outcomes. This means, even if paradoxical leaders serve as sensegivers to influence their subordinates, the sense-making by their followers may also fail when the organization’s identity is not incorporated into the individual’s self-concept. This result is also consistent with the assertion by Maitlis and Christianson (2014) that individuals are motivated to make changes in their own roles and actions when leaders are successful in influencing the sense-making of them. Taking together, our results suggest that with high levels of *Zhong Yong* thinking or high levels of organizational identification, paradoxical leadership may be sufficient for employees to fulfill their self-enhancement needs.

### Practical Implications

As organizational environments become more complex, fast-paced and competitive, organizations increasingly confront diverse paradoxical tensions. Leaders and employees seek coping strategies to deal with these paradoxical tensions. Our study may illuminate such strategies.

First, we highlight the importance for both leaders and employees of a paradox mindset to deal with contradictory demands. As prior research has suggested, coping with paradoxes is becoming an increasingly important skill (Zhang et al., 2015; Miron-Spektor et al., 2018). Second, our findings of the mediation effect of adaptability shows leaders that an effective way to increase performance is to improve employee adaptability. Developing paradoxical leadership may be an effective way for organizations to increase employee adaptability to promote positive outcomes. Finally, managers aiming to increase performance should be aware of different boundary conditions, such as culture. Employees who endorse traditional Chinese cultures will tend to understand leaders’ behaviors and reframe change in a positive way. For example, previous research has shown that *Zhong Yong* thinking guides employees in managing their meta-cognitions and preventing the disruptive effects of change (Pan and Sun, 2018). Managers should give such employees complex tasks and the discretion to act flexibly and autonomously. Moreover, rather than focusing on avoiding workplace contradictions, organizations should encourage greater organizational identification. Leaders’ sense-making and sensegiving is an effective route to identification (Gioia and Chittipeddi, 1991; Ashforth et al., 2008). Managers can enhance the prestige of the organization through the exchange of organizational information with employees, so that the identification of the organization becomes a contributor to the employees’ self-enhancement needs (Smidts et al., 2001).

### Limitations and Future Research

Admittedly, this study has several limitations. First, our multi-respondent cross-sectional data collection design by which we collected paradoxical leadership and employee adaptability at a single timepoint may cause common-method variance. Thus, future research should consider longitudinal or three-wave cross-sectional designs for data collection to replicate and extend the current research. Moreover, the CFI and TLI values in CFA are lower than 0.95. Future research should expand the sample size to improve the indices of model fit. Second, our research was based on a Chinese context, examining the influence mechanism of paradoxical leadership on performance outcomes from the perspective of individuals’ psychological processes. Future research could consider the differences between Chinese and Western situations when facing paradoxes. Third, we focused on individual differences and organizational context factors as boundary conditions that may affect the effectiveness of paradoxical leadership. Future research could explore other contextual variables that influence the effect of paradoxical leadership on employee outcomes.

## Data Availability Statement

The raw data supporting the conclusions of this article will be made available by the authors, without undue reservation.

## Ethics Statement

The studies involving human participants were reviewed and approved by Hubei University’s Ethics Committee. The patients/participants provided their written informed consent to participate in this study.

## Author Contributions

WZ developed the research model, analyzed the data, and co-drafted the manuscript. SL provided the constructive suggestions to the research design, collected the data, and co-drafted the manuscript. JL participated in the data analysis and edited the manuscript. QZ edited the manuscript. All authors listed have made a substantial, direct, and intellectual contribution to the work, and approved it for publication.

## Conflict of Interest

The authors declare that the research was conducted in the absence of any commercial or financial relationships that could be construed as a potential conflict of interest.

## Publisher’s Note

All claims expressed in this article are solely those of the authors and do not necessarily represent those of their affiliated organizations, or those of the publisher, the editors and the reviewers. Any product that may be evaluated in this article, or claim that may be made by its manufacturer, is not guaranteed or endorsed by the publisher.

## Footnotes

1
http://www.processmacro.org/download.html
